# Enhanced thermal conductivity of form-stable phase change composite with single-walled carbon nanotubes for thermal energy storage

**DOI:** 10.1038/srep44710

**Published:** 2017-03-16

**Authors:** Tingting Qian, Jinhong Li, Wuwei Feng, Hong’en Nian

**Affiliations:** 1 Beijing Key Laboratory of Materials Utilization of Nonmetallic Minerals and Solid Wastes, National Laboratory of Mineral Materials, School of Materials Science and Technology, China University of Geosciences (Beijing), Beijing 100083, P.R. China; 2 Key Laboratory of Comprehensive and Highly Efficient Utilization of Salt Lake Resources, Qinghai Institute of Salt Lakes, Chinese Academy of Sciences, Qinghai 266000, P.R. China.

## Abstract

A striking contrast in the thermal conductivities of polyethylene glycol (PEG)/diatomite form-stable phase change composite (fs-PCC) with single-walled carbon nanotubes (SWCNs) as nano-additive has been reported in our present study. Compared to the pure PEG, the thermal conductivity of the prepared fs-PCC has increased from 0.24 W/mK to 0.87 W/Mk with a small SWCNs loading of 2 wt%. SWCNs are decorated on the inner surface of diatomite pores whilst retaining its porous structure. Compared to PEG/diatomite fs-PCC, the melting and solidification time of the PEG/diatomite/SWCNs fs-PCC are respectively decreased by 54.7% and 51.1%, and its thermal conductivity is 2.8 times higher. The composite can contain PEG as high as 60 wt% and maintain its original shape perfectly without any PEG leakage after subjected to 200 melt-freeze cycles. DSC results indicates that the melting point of the PEG/diatomite/SWCNs fs-PCC shifts to a lower temperature while the solidification point shifts to a higher temperature due to the presence of SWCNs. Importantly, the use of SWCNs is found to have clear beneficial effects for enhancing the thermal conductivity and thermal storage/release rates, without affecting thermal properties, chemical compatibility and thermal stability. The prepared PEG/diatomite/SWCNs fs-PCC exhibits excellent chemical and thermal durability and has potential application in solar thermal energy storage and solar heating.

As known, latent heat storage fulfilled through a solid–liquid phase transformation is a particularly recommended technique^1^. However, the disadvantage for direct use of this kind of phase change materials (PCMs) relies in the leakage of the liquid phase above their melting temperature. The common approach to overcome this issue is to store the PCMs in the supporting materials for preparing form-stable PCM composites (fs-PCCs)^2^. Recently, diatomite has been popularly employed as PCM stabilization support due to its well-developed porosity and high surface area^3^.

It is of considerable interest to enchance the thermal conductivity of organic PCMs with various nano-additives^4^. This is because typical organic PCM, including sugar alcohol, paraffin, and alkane, has low thermal conductivity in the range of 0.1~1 W/mK, which has caused low store and release rates of the latent heat system^5^.

As known, carbon materials are the most popular additives for increasing the thermal conductivity of organic PCMs since only a small mass fraction is needed, and they have high thermal conductivity and low density. Recently, Nomura *et al*.^6^ proved that adding carbon nanofibres (CNFs) into the erythritol can obviously enhance the thermal properties of the erythritol. Besides, the thermal store rate of the solidification process was found to be increased with the CNFs increasement. Mehrali *et al*.^7^ dispersed the carbon nanosphere (CNS) into the stearic acid. The results showed that the thermal conductivity increased about 105% for the highest loading of CNS at 50 wt%.

A recent review on thermal conductivity enhancement of organic PCMs using the high conductive carbon nanotube has proved that the striking thermal conductivity improvement could be obtained using single-walled carbon nanotubes (SWCNs)^8^. SWCN is a fascinating family member of the carbon based nano-materials, which is characteristic for its outstanding thermal conductivity^9^.

Therefore, this study attempts to develop a novel form-stable PCM composite with high thermal conductivity. In our present study, PEG is the PCM, diatomite (Dt) was used as the supporting material, and SWCNs were employed as the high thermal conductive additive. The effective thermal conductivities of the prepared PEG/Dt/SWCNs fs-PCCs have been tested using the laser flash method and the microstructures were analyzed using transmission electron microscope (TEM). In addition, cyclic melting and solidification tests were performed to investigate the cyclic durability of the prepared fs-PCC. The resulting PEG/Dt/SWCNs fs-PCC is a potential solar thermal energy storage material for residential heating and domestic hot-water production.

## Results

### Characterization of Dt/SWCNs

**Figure 1:**
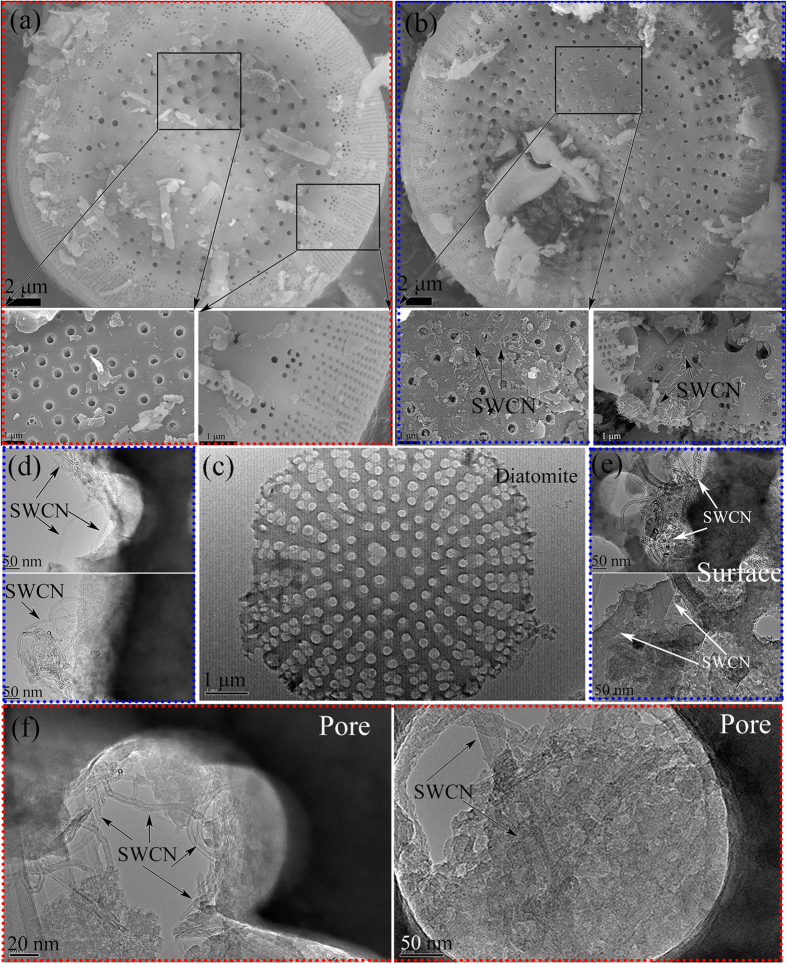
**SEM images of:** (**a**) diatomite; (**b**) Dt/SWCNs and **TEM images of:** (**a**) diatomite; (**b**–**d**) Dt/SWCNs.

Figure 1(a) shows the microstructure of the uncoated diatomite. The raw diatomite shows disk-shaped structure with well-developed porous structure and large void volume. The unique structure makes diatomite have high porosity and large specific surface area as expected. On account of this, diatomite has excellent adsorptive capacity and is a good support substance for PEG. Figure 1(b) demonstrates the SEM observations of diatomite after the immobilization of the SWCNs. As seen, the original geometry of the diatomite was not transformed after SWCNs impregnation and the porous structure of diatomite remains obviously visible. Besides, SWCNs are clearly found on the surface of diatomite.

Figure 1(c) shows the TEM image of the raw diatomite. It is found that there are numerous circular pores all over the disk. Figures 1(d,e) illustrate that the SWCNs are well and randomly dispersed on the surface of diatomite with the aid of ultrasonic dispersion. Besides, in Fig. 1(f), some well-dispersed SWCNs are observed to be inserted in the diatomite pores.

To further quantify the texture properties of the raw diatomite, Dt/SWCNs and PEG/Dt/SWCNs fs-PCC, three parameters including the specific surface area, pore volume and mean pore diameter were measured for comparison. The N_2_ adsorption–desorption isotherms and pore size distribution curves of diatomite and the Dt/SWCNs are presented in Fig. S1 (Supporting information) and the corresponding parameters are summarized in Table 1 for comparison. The table reveals that Dt/SWCNs has an average BET surface area of 28.57 m^2^/g, vs. 24.50 m^2^/g for the uncoated diatomite. Compared to the primary diatomite matrix, the BET areas and average pore diameters of Dt/SWCNs both increased by 17% and 26% after the SWCNs decoration, indicating that the pores of diatomite were not blocked. Besides, the particular web-like network of bundles and rough surface of SWCNs will contribute to the PEG adsorption. The high-resolution XPS spectra of the C 1 s region of Dt/SWCNs is shown in Fig. S2 (Supporting information). The peak position at 284.11 eV corresponds to the position of C 1 s of graphite. The result further proved that SWCNs have been successfully decorated on the diatomite surface.

### Characterization of PEG/Dt/SWCNs fs-PCC

**Figure 2:**
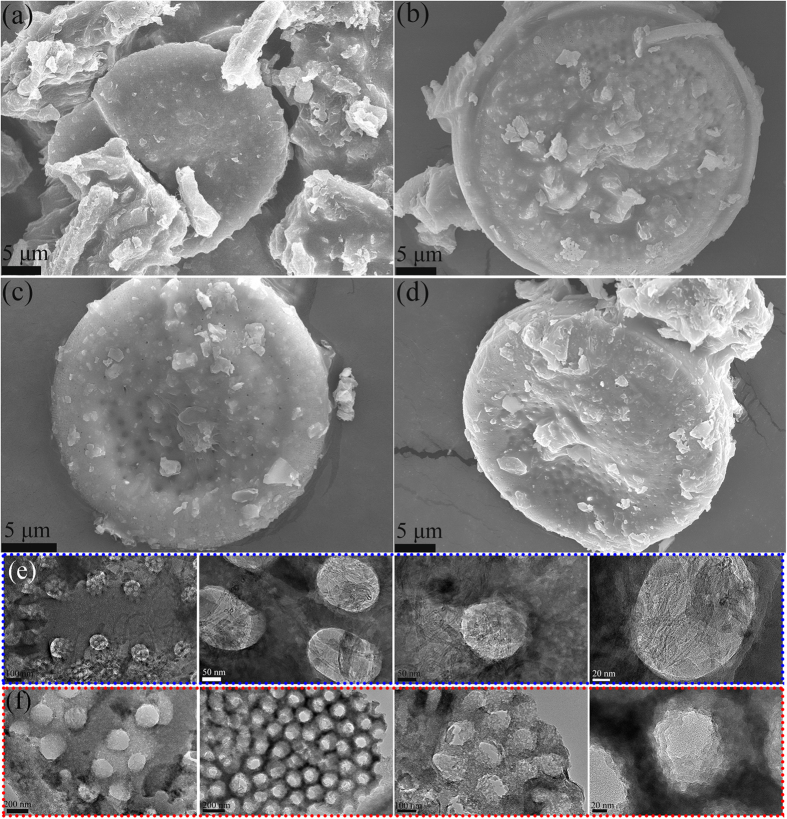
**SEM images of:** (**a–d**) PEG/Dt/SWCNs fs-PCC and **TEM images of:** (**e**) PEG/Dt/SWCNs fs-PCC; (**f**) PEG/Dt fs-PCC.

Figures 2(a–d) present the SEM observations of the PEG/Dt/SWCNs fs-PCC. Compared with the numerous pores observed in Fig. 1(b), there are only few visible pores left after PEG impregnation, indicating PEG was well impregnated into diatomite pores. SWCNs are not clearly found in the composite PCM. We speculated that SWCNs were covered by PEG chains. In our study, the maximum mass percentage of the PEG in the composite was as high as 60 wt%. For the composite prepared at the diatomite/PEG mass ratio of 70/30 and 60/40 (see Fig. S3 in Supporting information), the SWCNs can be clearly seen. It was noteworthy that the porous structure of diatomite can effectively provide the mechanical support for the fs-PCC and can prevent the leakage of molten PEG because of the effect of capillary forces and surface tension between PEG molecules and diatomite pore walls.

The N_2_ adsorption–desorption analysis results of Dt/SWCNs and the PEG/ Dt/SWCNs are summarized in Table 1 for comparison. Compared with the BET area of Dt/SWCNs, the BET area of the fs-PCC decreases sharply with the PEG impregnation, indicating the full adsorption of PEG into pore structure of diatomite.

Figure 2(e) illustrates the TEM images of the prepared fs-PCC. PEG is well impregnated into the diatomite pores. Besides, a random distribution of SWCNs on the surface and in the pores of diatomite can be clearly found. They are randomly aligned with a diameter of approximately 20~40 nm and a length of several micrometers. Another four TEM images of the PEG/Dt are shown in Fig. 2(f) for comparison. Similarly, PEG is dispersed in the porous network of the diatomite. But no trace of SWCNs is found. However, some pores that are not full of PEG are clearly seen in Fig. 2(e,f). We guessed that some PEG lost in the distilled water under the ultrasonic treatment before TEM testing. Usually, the ultrasonic treatment is applied for preparation of dispersal TEM specimen.

**Figure 3:**
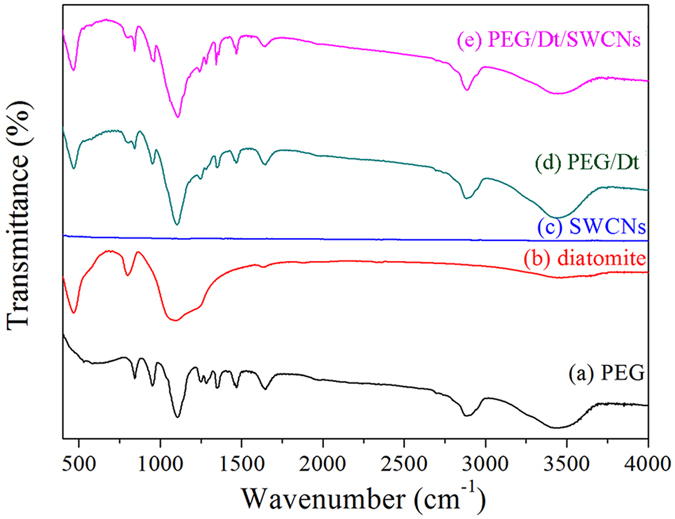
FT-IR spectra of PEG, diatomite, SWCNs, PEG/Dt and PEG/Dt/SWCNs fs-PCC.

Figure 3 shows the FT-IR spectra of different samples. In Fig. 3(a), the stretching vibrations found at 844, 948 and 2879 cm^−1^ belong to the –CH_2_ of PEG. The stretching vibration of C–O is observed at 1106 cm^−1^. Besides, a broad absorption peak centered at 3442 cm^–1^ can be assigned to the stretching vibration of the single bond OH groups. These characteristic absorption peaks are all in good agreement with those of the pure PEG. In Fig. 3(b), the broad absorption peak of diatomite at 3446 cm^−1^ represents the stretching vibration of the intermolecular single bond OH groups. The peaks at 1107 and 466 cm^−1^ can be assigned to asymmetric and symmetric Si–O–Si stretching vibrations of the diatomite, respectively. Consistent with the previously reported results^10^, no detectable transmission band is found for the SWCNs in the wavenumber range covered in this study, as shown in Fig. 3(c). In Fig. 3(d), all the main absorption peaks of PEG and diatomite appear as predicted. No obvious new peak is seen, indicating that there was no chemical interaction between PEG and diatomite. In Fig. 3(e), a comparison between PEG/Dt and PEG/Dt/SWCNs indicates that the peaks in the PEG/Dt/SWCNs spectrum at 3444, 2883, 1106, 960, 840, 800, and 466 cm^−1^ appear at the same positions as those of the PEG/Dt spectrum. The maxima of both spectra occurred in the middle infrared region (4000–400 cm−1), indicating that there was no chemical interaction between PEG/Dt and SWCNs, while the combination of both was possible only by physical adsorption.

### Effect of SWCNs on the thermal behavior of PEG/Dt/SWCNs fs-PCC

**Figure 4:**
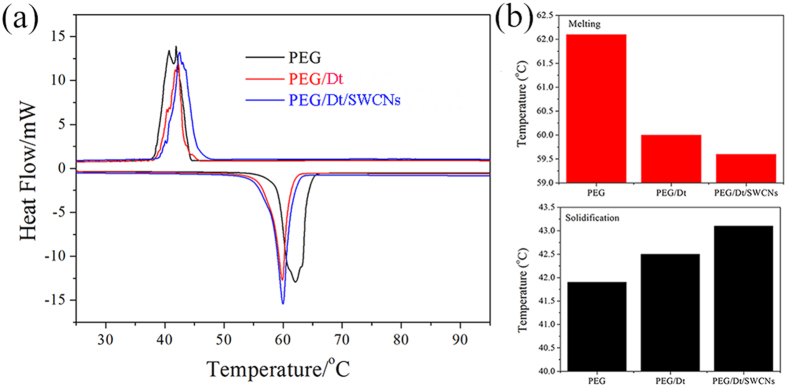
(**a**) DSC curves and (**b**) melting and freezing temperatures of PEG, PEG/Dt and PEG/Dt/SWCNs.

Figure 4(a) shows the typical DSC thermograms of the PEG/Dt and PEG/Dt/SWCNs fs-PCC. The corresponding data collected from the DSC tests are summarized in Table 2. The DSC results suggest that, both the composites while undergoing cyclic melting and freezing processes have exhibited only single peak, which conformed to the liquid-to-solid phase transition. The PEG, PEG/Dt and PEG/Dt/SWCNs presented latent heats of melting of 188.1 J/g, 104.6 J/g, and 109.8 J/g, respectively, while latent heats of freezing of 177.2 J/g, 97.6 J/g and 103.8 J/g, respectively. The values for latent heat capacity of the PEG/Dt and PEG/Dt/SWCNs were nearly 55.6% and 58.3% than that of the pure PEG, respectively. The measured latent heats of melting and freezing of the two fs-PCCs were close to the values calculated by multiplying the mass ratio of the PEG in the composites, and their phase change enthalpies^11^. Moreover, it is obvious that for the two composites, the angles on melting point are shaper and the endothermic peaks are narrower than that of the PEG. This phenomenon could be ascribed to the porous diatomite network in the composites that provided heat conduction path in the PEG and consequently accelerated the phase change speed of the composites.

In Fig. 4(b), for the two PEG fs-PCCs, the freezing temperature is observed to slightly shift towards a higher temperature, whereas the melting temperature decreases slightly. For example, the melting temperature of the PEG/Dt/SWCNs is 59.6 °C, and is slightly lowered for the presence of SWCNs while compared to the PEG/Dt composite. In addition, similar trends can be seen for the freezing temperature of the two types of PEG-based composites. The addition of SWCNs is shown to slightly raise the freezing temperature. Irrespective of experimental errors, the observed changes in the phase transition temperatures may be attributed to the SWCNs additive in the PEG/Dt/SWCNs composite that provides heat conduction path in the PEG/diatomite and consequently accelerates the phase change speed of the composite.

### Thermal and shape stability of PEG/Dt/SWCNs fs-PCC

**Figure 5:**
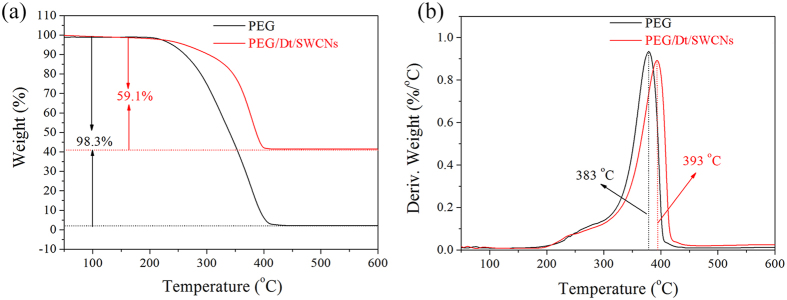
TGA curves and the corresponding DTG thermograms of PEG and PEG/Dt/SWCNs fs-PCC.

Figure 5 shows TGA curves and DTG thermograms of the PEG and PEG/Dt/SWCNs up to 600 °C. The TGA curve of the prepared fs-PCC almost overlaps with that of the PEG at low temperature zone, indicating the fs-PCC had good thermal stability before 250 °C. In the temperature range of 250 to 400 °C, there is an obvious degradation step for PEG and composite, attributing to the complete decomposition and the evaporation of the organic alcohol compounds. Only 1.7% of residue is obtained at 600 °C for PEG, while the fs-PCC has a higher residue of 40.9 wt%. The DTG curves exhibit a narrow peak corresponding to the temperature at the rapid weight loss of the PEG. It is reasonable to believe that the encapsulated PEG must first break through the pores of diatomite during heating process and then can evaporate out. The diatomite pores are rigid enough to prevent the impregnated PEG from evaluating at normal boil point, thus improving the degradation temperature of the PEG fs-PCC. It is evident from the result that, the PEG/Dt/SWCNs was more stable and exhibited increased resistance to the heat than the pure PCM.

The form-stable property of the PEG/Dt/SWCNs fs-PCC is shown in Fig. 4S. The prepared fs-PCC maintains solid unlike pure PEG, which displayed solid-to-liquid phase transition. Besides, no liquid PEG trace was found throughout the entire heating process even when the temperature was 90 °C, indicating that the PEG/Dt/SWCNs hybrid had excellent form-stable property. In addition, the mass loss during the melting process can be neglected. Based on the analysis above, the prepared PEG/Dt/SWCNs fs-PCC is quite stable in both thermal and shape behavior.

### Thermal conductivity of PEG/Dt/SWCNs fs-PCC

**Figure 6:**
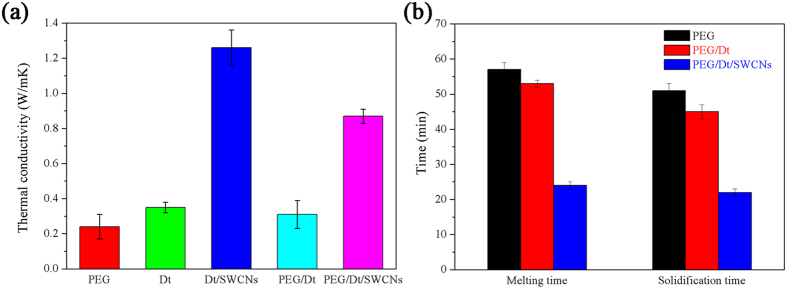
(**a**) Thermal conductivity and (**b**) the melting and solidification times of PEG, PEG/Dt and PEG/Dt/SWCNs.

Figure 6(a) shows the measured thermal conductivities of different samples. The thermal conductivity of PEG is determined to be 0.24 W/mK, whereas for the PEG/Dt/SWCNs, the thermal conductivity has increased to 0.87 W/mK. The striking enhancement, as high as 260%, was mainly ascribed to the porous diatomite and high thermal conductive and well-dispersed SWCNs. Besides, in comparison with PEG/Dt, the thermal conductivity of PEG/Dt/SWCNs is significantly boosted with the enhancing level as high as 180%. Addition of the SWCNTs is the major attributed reason. Table 3 presents the comparison of the PEG/Dt/SWCNs fs-PCC with results of other diatomite supported fs-PCCs in literature. SWCN was found to be firstly used to improve the thermal conductivity of diatomite-supported fs-PCM so far. It is found that SWCNs with small mass fraction can greatly improve heat transfer efficiency because of the high thermal conductivity of SWCNs and the increase margin is the largest to the best of our knowledge without affecting its thermal properties. For example, compared to the paraffin/diatomite using multi-walled carbon nanotubes (MWCNTs) as additive^12^, both the increase margin of thermal conductivity and thermal storage capacity are competitive and preferred. Therefore, the prepared PEG/Dt/SWCNs fs-PCC has an important potential application in thermal energy storage system.

The melting and solidification performances of PEG, PEG/Dt and PEG/Dt/SWCNs were conducted (Supporting information). In our study, the melting time is estimated as a time elapsed from the same initial temperature of 25 °C to over melting point of the PEG (80 °C); and accordingly the solidification time is determined as a time consumed from 80 °C to 25 °C. Figure 6(b) compares the time that required for the melting and solidification performance of PEG and the prepared fs-PCCs. From Fig. 6(b), 53 min and 45 min were required for the melting and solidification process of PEG/Dt fs-PCC, respectively. However, the melting process took only 24 min and the solidification process took 22 min for PEG/Dt/SWCNs fs-PCC. The decrease in the melting and solidification times of the PEG/Dt after SWCNs loading can be ascribed to the large increase in the thermal transfer rate during heating and cooling cycles.

### Thermal reliability of PEG/Dt/SWCNs fs-PCC

**Figure 7:**
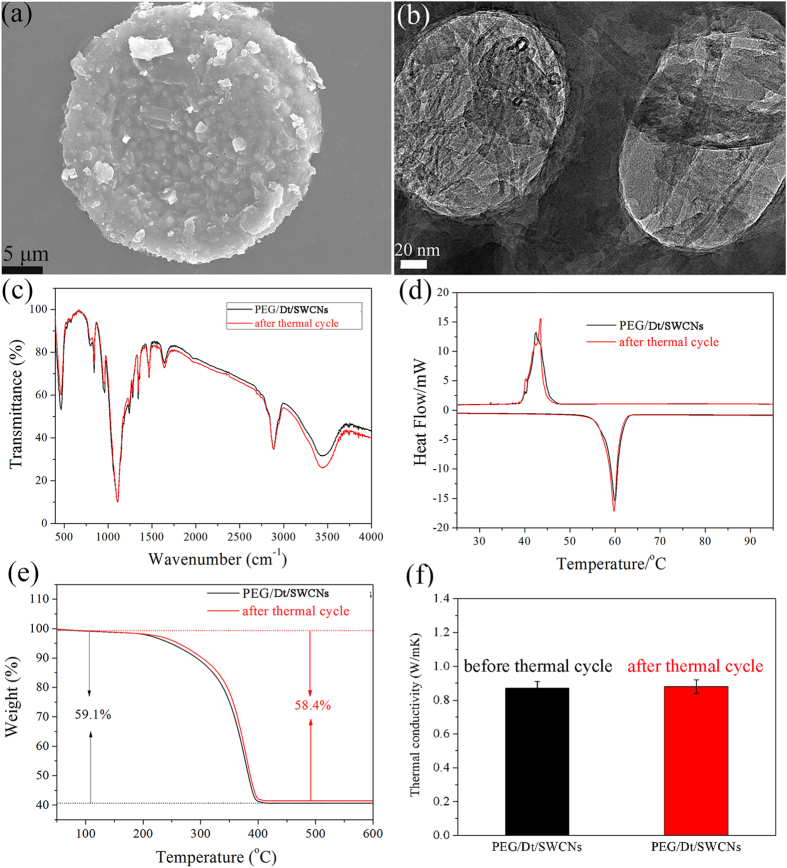
The PEG/Dt/SWCNs fs-PCC after 200 thermal cycle: (**a**) SEM image; (**b**) TEM image; (**c**) FTIR spectra; (**d**) DSC curves; (**e**) TGA curve; (**f**) Thermal conductivity measurement.

As known, a PCM should maintain the same or almost the same thermal, chemical and physical properties even after a number of repeated melting/freezing cycles, which is known as thermal reliability. Therefore, a 200 thermal cycle test was performed for the prepared PEG/Dt/SWCNs and no leakage of the liquid PEG was found. SEM image of PEG/Dt/SWCNs after the thermal cycle is shown in Fig. 7(a). PEG is well adsorbed into the diatomite pores. In addition, SWCNs are clearly seen in the diatomite pores as shown in TEM image of Fig. 7(b). Figure 7(c) compares the FT-IR spectra of the fs-PCC before and after the cycle test. No obvious difference can be seen. The chemical structure of shape-stabilized composite PCM was not affected by thermal cycle. Chemical degradation did not occur during thermal cycling process.

Figure 7(d) compares the DSC curve of the prepared fs-PCC before and after thermal cycle. The melting temperature changed as 0.3 °C, and the solidification temperature changed as 0.5 °C. In addition, the latent heat enthalpy of the melting changed as 3.3% and the latent heat enthalpy of solidification changed by 4.2%. Figure 7(e) shows the TGA curves of the fs-PCC before and after thermal cycle. The almost overlapped two curves indicated that the fs-PCC had excellent thermal stability even after thermal cycle. Figure 7(f) compares the thermal conductivity measurement of fs-PCC before and after cycle test. The value keeps the same. It can be concluded that the prepared PEG/Dt/SWCNs fs-PCC had excellent thermal reliability in terms of the negligible changes in its phase transition temperatures, latent heats, thermal stability and conductivity.

## Discussion

Although diatomite-supported fs-PCCs have been extensively studied, we observed that the diatomite particles employed were raw materials without any further modification, ultimately resulting in the low thermal conductivity, which will in turn reduce energy-storing efficiency. Our solution to this issue is to decorate the raw diatomite with high thermal-conductive nano-additives before use, making it more qualified to be a PCM carrier. As known, carbon materials are the preferred additives for enhancing the effective thermal conductivity since only a small mass fraction is needed due to the high thermal conductivity and low density. In our study, we firstly reported a novel shape-stabilized and high thermal-conductive diatomite-supported composite PCM with SWCNs additive. SWCN has been firstly reported to be used to improve the thermal conductivity of diatomite-supported fs-PCM so far. But above all, we compared the thermal conductivity of the prepared fs-PCC with that of other diatomite-supported ss-PCC, and found that the increase margin is the largest in literature to the best of our knowledge without affecting its thermal properties, chemical compatibility and thermal stability. Moreover, in other papers, the increase in thermal conductivity is based on the consumption of phase change enthalpy, but our paper, this phenomenon does not occur. The results obtained from the comparison distinguish this work from previous reports.

The particular web-like and intertwined network of SWCNs as shown in SEM and TEM observations could provide a fast heat conduction path throughout the fs-PCC, promoting the heat transfer rate of the PEG. In this way, the phase change speed of the fs-PCC would be greatly accelerated, and thus resulting in faster melting and freezing of the sample. Besides, the melting temperature of the prepared fs-PCC decreases with the presence of SWCNs, whereas the freezing temperature increases, contributing to a lower degree of supercooling and indicating that the SWCNs additive could further enhance the heat transfer performance.

As known, carbon materials are promising for improving the thermal conductivity of PCM, thus, a direct comparison of their performance has been discussed in our present study. Fan *et al*.^13^ once proved that the thermal conductivity of the composite PCMs was found to increase with the presence of carbon nanofillers, and the relative enhancement strongly depends on the size and shape of the nanofillers. The crabon nanotubes (CNTs) caused much more increase in thermal conductivity than what the carbon nanofibers (CNFs) did. For the two types of wire-shaped CNTs and CNFs, the thermal conductivity enhancement seems to become less pronounced with increasing the size. However, carbon nanosphere (CNS) is less effective than the two wire-shaped carbon fillers in improving the thermal conductivity due to its relatively low thermal conductivity and small specific surface area^7^. The detailed values of thermal conductivity enhancement by CNFs^13^ and CNS^7^ have been listed in Table S1 in the supporting material for comparison. Thus, in our present study, SWCN was chose due to its particular web-like and intertwined network and extremely high thermal conductivity obtained from Table S1.

## Conclusion

Our study has reported a high thermal conductive PEG/Dt/SWCNs form-stable composite. SWCNs were randomly distributed on the surface of diatomite whilst retaining its porous structure (SEM, TEM, BET and XPS results). With a small SWCN loading of 2 wt% a strikingly high, 260% enhancement is obtained in the thermal conductivity, compared to the pure PEG. In addition, its thermal conductivity was 2.8 times higher than that of the PEG/Dt fs-PCC. The decreased melting and solidification times have further confirmed this result. These values remained unchanged even after a 200 thermal cycle test. The maximum load of PEG can reach as high as 60 wt%. DSC analysis showed that the fs-PCC melt at 59.6 °C with a latent heat of 109.8 J/g and solidified at 43.1 °C with a latent heat of 103.8 J/g. The melting temperature of the fs-PCC was marginally lowered while the freezing temperature was slightly raised due to the presence of SWCNs additive. It is easy to find that SWCNs had no significant effect on the latent heat of composite PCM. SEM and TEM results proved that PEG was well adsorbed even after the thermal cycle. The excellent chemical compatibility, thermal stability and durability of the ss-PCC were also beneficial for thermal energy storage applications such as building energy conservation and domestic hot water and heating systems.

## Methods

### Preparation of Dt/SWCNs

**Figure 8:**
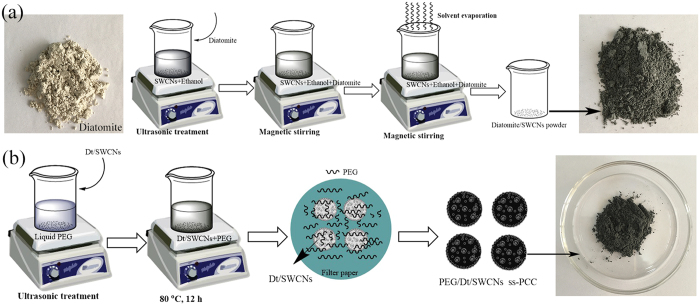
Schematic diagram of the preparation process of: (**a**) Dt/SWCNs; (**b**) PEG/Dt/SWCNs fs-PCC.

Raw diatomite was crushed and treated with 4 M H_2_SO_4_ at 80 °C for 12 h, rinsed with distilled water until pH 7 and dried overnight^14^. Single-walled carbon nanotube was supplied by Xilong Chemical Reagent Beijing Co., Ltd. China. Figure 8(a) illustrates the schematic process for the SWCNs decoration on diatomite. Firstly, a desirable amount of SWCNs was dispersed in ethanol using the intensive ultrasonic probe equipment. Then diatomite was added with a further continuously stirring using the magnetic stirrer equipment. To ensure diatomite was uniformly decorated with the dispersed SWCNs, the Dt/SWCNs ethanol suspension was further continuously stirred for 2 h using magnetic stirrer equipment. Finally, a dry mixture of diatomite and the dispersed SWCNs was received after overnight drying. Compared to the yellow-tinted color of diatomite, Dt/SWCN has a deep dark color due to the incorporation of 2 wt% SWCNs.

### Preparation of PEG/Dt/SWCNs fs-PCC

PEG (Mw = 6000) was provided by Beijing Chemical Reagent Co., Ltd. The PEG/Dt/SWCNs fs-PCC was prepared with a facile blending and impregnation method as shown in Fig. 8(b). Dt/SWCNs powder was first infused in the melt PEG with the aid of sonication. Then the mixture was kept at 80 °C for 12 h. Finally, the mixture was filtrated and dried at 80 °C for 12 h to remove the liquid PEG from Dt/SWCNs. Only the sample that has the largest adsorption ratio and maintains its original shape without any leakage when heated above the melting point of PEG is identified as the form stable composite. In present study, the maximum mass ratio of PEG in the fs-PCC is 60 wt%.

### Analysis methods

The microstructures of the samples were examined by scanning electron microscopy (SEM, S-4800, Hitachi, Japan) and transmission electron microscopy (TEM, JEOL JEM-2010, Japan, accelerating voltage 200 KeV). The specific surface area and pore volume of diatomite were determined by a N_2_ adsorption analyzer (Quantachrome Instruments, US). A PHI-5300 X-ray photoelectron spectroscopy system (XPS, Perkin Elmer, America) was used to confirm the presence of the carbon membrane loaded on the diatomite surface. The chemical compatibility of composite PCMs was obtained via Fourier transform infrared spectroscopy (FT-IR, Model Frontier). The stability of the fs-PCC was explored through the thermo-gravimetric analysis (TGA, Q50) with a heating rate of 10 °C/min. Differential scanning calorimeter (DSC, Q2000) was used to measure solid–liquid phase change temperatures and latent heat capacities of the fs-PCMs. All the operation was conducted under a nitrogen atmosphere. The phase change temperatures and latent heats of the prepared fs-PCC were obtained at 5 °C/min in the temperature range of 25~80 °C. The hot disk thermal constant analyzer (TPS2500) was employed to measure thermal conductivity. All the values are measured at room temperature. The thermal cycle test of the prepared ss-PCC was carried out by placing the sample on a filter paper. It was then placed in a thermostatic chamber and maintained at 80 °C. The ss-PCC was exposed alternately to room temperature and to 80 °C. During this process, the filter paper was changed after every 10 cycles, with each cycle including a melting and solidification step. The ss-PCC was tested for 200 cycles.

## Additional Information

**How to cite this article**: Qian, T. *et al*. Enhanced thermal conductivity of form-stable phase change composite with single-walled carbon nanotubes for thermal energy storage. *Sci. Rep.* **7**, 44710; doi: 10.1038/srep44710 (2017).

**Publisher's note:** Springer Nature remains neutral with regard to jurisdictional claims in published maps and institutional affiliations.

## Supplementary Material

Supplementary Information

## Figures and Tables

**Table 1:** Textural properties of diatomite, Dt/SWCNs, and PEG/Dt/SWCNs before and after thermal cycle test.

Results	Samples			
	Dt	Dt/SWCNs	PEG/Dt/SWCNs	PEG/Dt/SWCNs after thermal cycle
Specific surface area (m^2^/g)	24.50	28.57	2.836	2.751
Pore volume (cc/g)	0.042	0.037	0.013	0.013
Mean Pore Diameter (nm)	3.321	4.201	1.231	1.300

**Table 2:** Thermal characteristics of PEG and the prepared fs-PCCs.

Samples	PEG Mass Ratio (wt%)	Melting Process		Solidifying Process	
		H_M_(J/g)	T_M_(°C)	H_S_(J/g)	T_S_(°C)
PEG	100	188.1	62.1	177.2	41.9
PEG/Dt	57	104.6	60.0	97.6	42.5
PEG/Dt/SWCNs	60	109.8	59.6	103.8	43.1
fs-PCC after thermal cycle	60	106.2	59.3	99.4	42.7

**Table 3:** Comparison of the diatomite supported fs-PCCs in literature.

PCM	Additive	Mass fraction	Melting Process		Solidification Process		Thermal conductivity (W·m^−1^·K^−1^)	Ref.
			H_M_ (J/g)	T_M_ (°C)	Hs (J/g)	T_S_ (^o^C)		
Paraffin	Multi-wall carbon nanotubes	0.26%	89.40	27.12	89.91	26.50	1.7 (42%↑)	12
Hexadecane	Exfoliated graphite nanoplatelet	5%	120.8	22.09	120.1	17.14	0.42	15
PEG	Expanded graphite	10%	189.30	59	168.70	41	0.71	16
PEG	Expanded graphite	10%	79.64	27.36	76.21	31.12	0.67	3
PEG	Nano-Ag	7.2%	111.3	59.45	102.4	41.02	0.82	17
PEG	SWCNs	2%	109.8	59.6	103.8	43.1	0.87 (180%↑)	present study
