# Supplementary material for: Enhanced thermal conductivity of form-stable phase change composite with single-walled carbon nanotubes for thermal energy storage
Source: Sci Rep. 2017 Mar 16;7:44710. doi: 10.1038/srep44710 (PMC5353756; doi:10.1038/srep44710)
Supplement: Supplementary Information [file srep44710-s1.pdf]

Enhanced thermal conductivity of form-stable phase change composite with single-walled carbon nanotubes for thermal energy storage

Tingting Qian[1],* Jinhong Li[1],* Wuwei Feng[1], Hong'en Nian[2]

[1] Beijing Key Laboratory of Materials Utilization of Nonmetallic Minerals and Solid Wastes, National Laboratory of Mineral Materials, School of Materials Science and Technology, China University of Geosciences (Beijing), Beijing 100083, P.R. China, [2] Key Laboratory of Comprehensive and Highly Efficient Utilization of Salt Lake Resources, Qinghai Institute of Salt Lakes, Chinese Academy of Sciences.

Results

Characterization of Dt/SWCNs

The nitrogen adsorption–desorption isotherm of the diatomite is characterized as a type II isotherm with an H3 hysteresis loop. The hysteresis is associated with the filling and emptying of the mesopores by capillary condensation, indicating the existence of mesopores in the diatomite. Besides, the sharp increase in the nitrogen adsorbed amount near the relative pressure of 1 corresponds to the adsorption by macropores. Such a feature suggests the co-presence of macropore and mesopore structures, as shown in SEM and TEM images. In addition, the N_2 adsorption–desorption isotherms of the two samples are similar, indicating that the microstructure of the diatomite is maintained after SWCNs decoration.

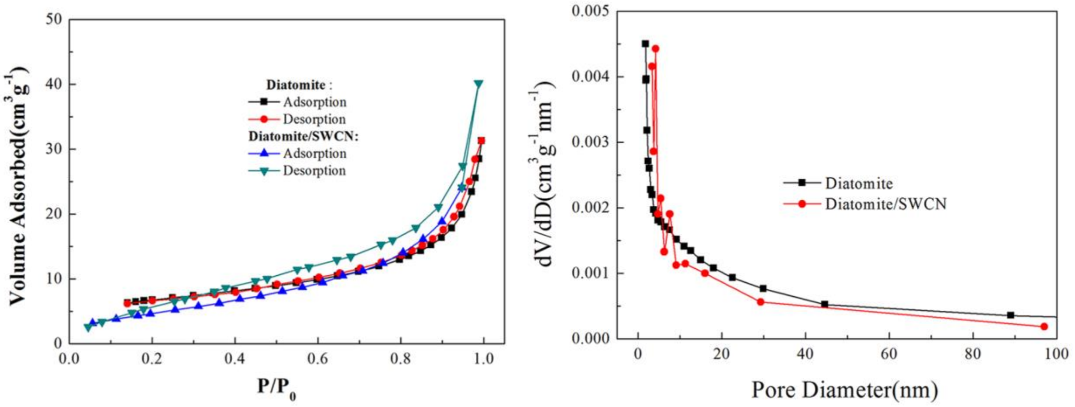

Fig. S1 N_2 adsorption–desorption isotherms and pore size distributions of diatomite and Dt/SWCNs

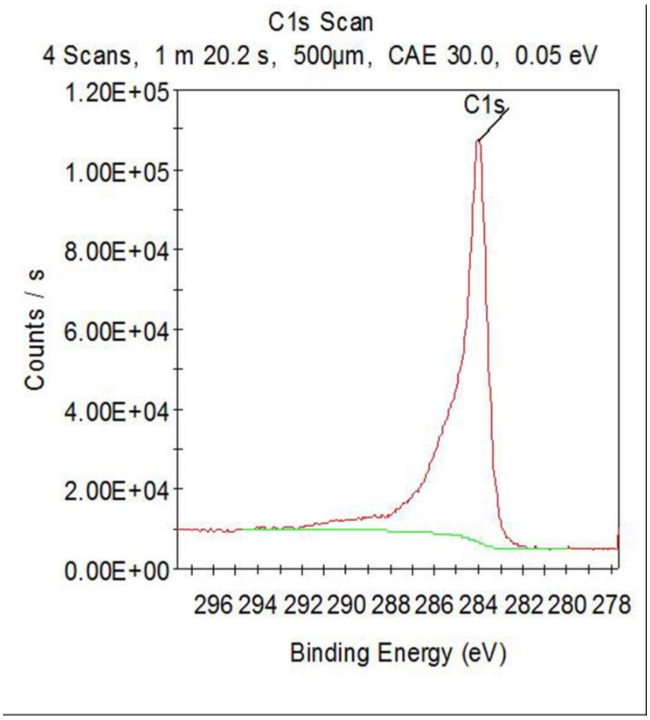

Fig. S2 High-resolution XPS spectrum of C 1s in Dt/SWCNs

Characterization of the prepared PEG/Dt/SWCNs ss-PCC

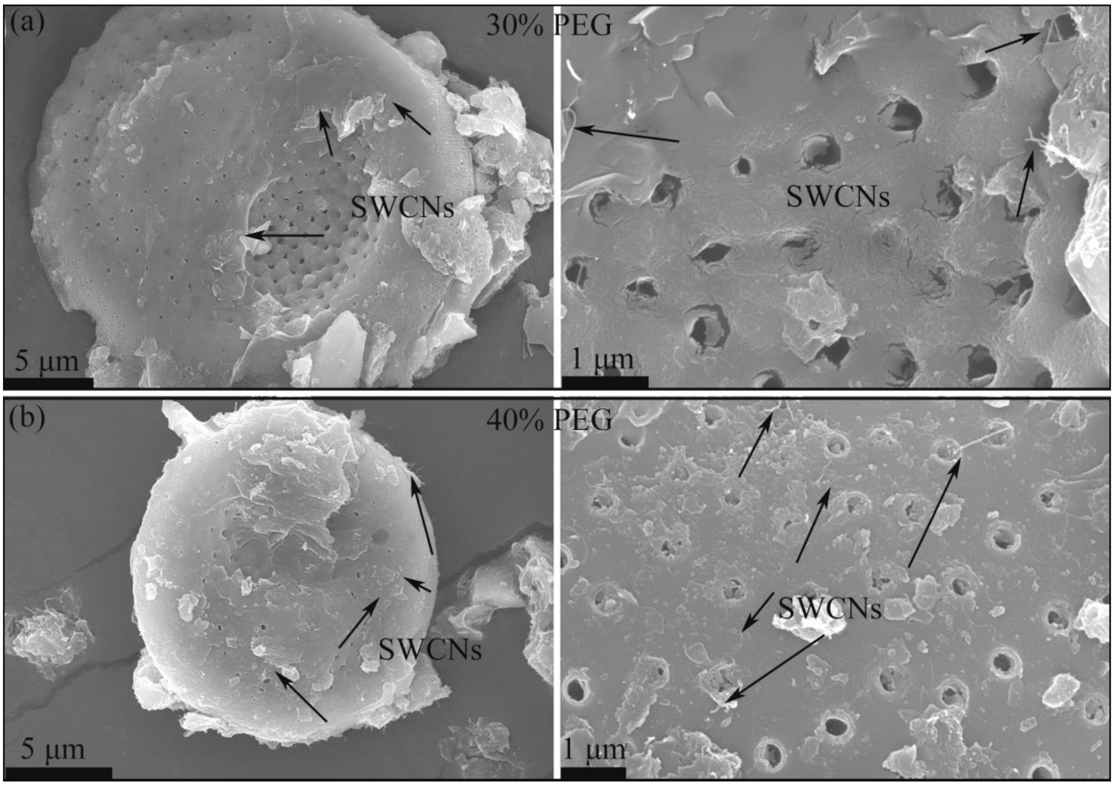

Fig. S3 SEM images of ss-PCC prepared at the diatomite/PEG mass ratio of 70/30 and 60/40

Thermal and shape stability of the prepared PEG/Dt/SWCNs ss-PCC

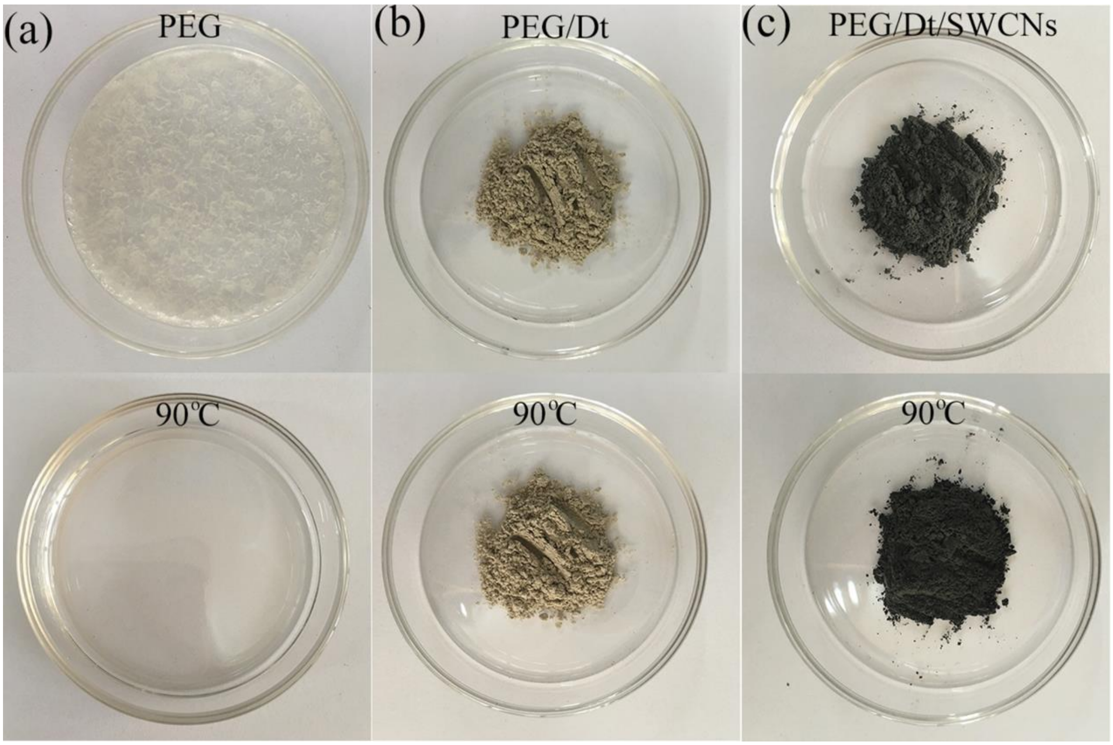

Fig. S4 Photos of PEG, PEG/Dt, and PEG/Dt/SWCNs at 25 and 90 °C

Thermal conductivity of the prepared PEG/diatomite/SWCNs ss-PCM

To determine the melting and freezing characteristics of the prepared PEG/Dt and PEG/Dt/SWCNs composite PCMs, 20 g PEG, PEG/Dt and PEG/Dt/SWCNs were placed into three glass vessels, respectively. A thermometer with the temperature accuracy of 0.5 °C was placed in the center of the vessels. The three testing vessels were put into a drying oven at 80 °C for complete melting and then immediately put into the water bath with a constant temperature of 25 °C for the solidification process. The temperature variations of the two composite PCMs during melting and solidifying periods were measured and recorded.

Discussion

Table S1 Comparison of the thermal conductivity enhancement by CNS and CNFs

PCM	Additive	Mass fraction	Thermal conductivity (W m^{-1} K^{-1})	Increased (%)	Ref.
Stearic acid	CNS	0	0.21	----	[7]
		11%	0.301	43%	
		20%	0.381	81%	
		33%	0.415	98%	
		50%	0.431	105%	
Paraffin	CNFs	0	0.263	----	[17]
		5%	0.305	16%	
PEG	SWCNs	2%	0.87	260%	Present study

[7] Mehrali M., Latibari S. T., Mehrali M., Mahlia T. M. I. & Metselaar H. S. C. Effect of carbon nanospheres on shape stabilization and thermal behavior of phase change materials for thermal energy storage. *Energy Convers.* Manage. **88**, 206–213 (2014).

[17] Fan L.W., Fang X., Wang X., Zeng Y. & Xiao Y.Q. et al. Effects of various carbon

nanofillers on the thermal conductivity and energy storage properties of paraffin-based nanocomposite phase change materials. *Appl. Energy* **110**, 163–172 (2013).
